# Plasticity of symbiotroph-saprotroph lifestyles of *Piloderma croceum* associated with *Quercus robur* L.

**DOI:** 10.1038/s42003-025-08762-w

**Published:** 2025-09-16

**Authors:** Witoon Purahong, Benjawan Tanunchai, Li Ji, Hagen Stellmach, Boaz Hilman, Ernst-Detlef Schulze, Bettina Hause, Mika Tarkka, François Buscot, Sylvie Herrmann

**Affiliations:** 1 UFZ-Helmholtz Centre for Environmental Research, Department of Soil Ecology, Halle (Saale), Germany; 2 Bayreuth Center of Ecology and Environmental Research (BayCEER), University of Bayreuth, Bayreuth, Germany; 3 School of Forestry, Central South University of Forestry and Technology, Changsha, PR China; 4 Leibniz-Institut für Pflanzenbiochemie, Halle, Germany; 5 Max Planck Institute for Biogeochemistry, Biogeochemical Processes Department, Jena, Germany; 6 German Centre for Integrative Biodiversity Research (iDiv), Halle-Jena-Leipzig, Leipzig, Germany

**Keywords:** Microbial ecology, Fungal ecology, Forest ecology

## Abstract

Besides their symbiotic association with tree rootlets, ectomycorrhizal (EM) fungi have been commonly detected in nature in deadwood and plant debris of various tree species. However, their potential dual roles as symbiotrophs and saprotrophs are still debated. Here, we provide evidence from a series of experiments on the plasticity of symbiotrophic-saprotrophic lifestyles of the ectomycorrhizal fungus *Piloderma croceum* associated with *Quercus robur* L. Specifically, we find that *P. croceum* efficiently colonizes deadwood of oak in an experimental system without living oak. Results based on the productions of hydrolytic enzymes and corticrocin as well as the ^14^C content in deadwood and mycelium of *P. croceum* demonstrate its capability of wood decomposition and assimilation of C from the decomposing wood. Our results also show that in presence of wood pieces colonized by saprotrophic mycelium of *P. croceum*, the roots of oak plants develop true EM symbiosis with Hartig net formation. Collectively, our results indicate a role for mycelium growing in deadwood as an underestimated EM fungus propagule bank, suggesting that deadwood and other decomposing plant material may indirectly influence the productivity of forest ecosystems by contributing to the recruitment of mycorrhizal fungi, thereby enhancing plant nutrient acquisition.

## Abstract (web summary)

Evidence is provided for plasticity between symbiotrophic and saprotrophic lifestyles of the ectomycorrhizal (EM) fungus *Piloderma croceum* associated with *Quercus robur* L., indicating a role for deadwood as an underestimated EM fungus propagule bank.

## Introduction

Ectomycorrhizal fungi (EM fungi) play a central role in providing trees with important nutrients especially in boreal and temperate but also in some subtropical and tropical forest ecosystems^1^. The existence and functioning of EM fungal mycelia determine plant growth and productivity in the forest ecosystems and contribute to the large global pool of carbon in soils^1,2^. The mycelia of EM fungi extend from different individual host tree species and form common mycorrhizal networks in soils, which influence plant nutrient and carbon acquisition at ecosystem level^3^. These mycelia can act as propagules for EM fungi to colonize seedlings and stimulate forest regeneration^3^. In areas where mycorrhizal networks are not available or poorly formed, EM fungal spores in soils can be considered an important source of propagules^4^. EM fungal spores may persist in the soil for decades before colonizing plant roots^5^. Extramatrical mycelium and spores in soils are thus considered as the major propagule banks of EM fungi^3^, but it is currently unknown whether EM fungi colonized plant debris could also serve as propagule banks.

Recent experimental evidence, based on high-throughput sequencing, has demonstrated that deadwood harbors diverse EM fungal communities^6–8^. EM fungi colonize deadwood and use this niche, among others (litter and living roots). EM fungi have evolved on repeated independent occasions among clades of initially humus and wood saprotrophic fungi, so that EM fungal taxa are spread across the classification of Asco- and Basidiomycota, where they still neighbor some saprotrophic sister taxa within clades^9,10^. EM fungi can compete with saprotrophs in deadwood. Their competitiveness depends on priority effects (or community assembly history), depending on their early arrival to the substrate or the wood decay stage^8,11^. Although EM fungi can also be detected at early wood decay stages in some temperate and subtropical tree species, they dominate during the late decomposition stages^6,8,12^. Some EM fungi have been suggested to have proteolytic activity via the secretion of relevant enzymes that support the decomposition of dead biomass^13,14^. Several studies have demonstrated the potential of EM fungi to degrade complex plant polymers, including cellulose, hemicellulose, pectin, and lignin^15,16^. However, the enzymatic machinery for the decomposition of these polymers is strongly reduced in EM fungi^15,17,18^. Some EM fungi can oxidize organic matter, either by Fenton chemistry, such as *Paxillus involutus* and *Piloderma croceum*^19^, or by using peroxidases, such as *Cortinarius* spp.^20^. It has been suggested that these activities serve to obtain nitrogen from organic sources^21^. The conservation level of enzymes for plant polymer decomposition is particularly high in *P. croceum*, whose genome contains 52, 18, and 38 genes related to cellulose/hemicellulose, pectin, and lignin decomposition, respectively^17^. Such carbon degrading enzyme activities can serve to release glucose and other small carbon compounds and molecules from complex organic sources^22,23^ which can be utilized by *P. croceum*. Therefore, decomposing plant materials may promote ectomycorrhizal establishment and propagation of *P croceum* in temperate forest ecosystems^24^. It has been hypothesized to be a potential ectomycorrhizal propagule bank in forest ecosystems^6,25^. Although some EM fungi might have evolved from either saprotrophic or endophytic fungi, their evolution via saprotrophic fungi has been more common^26^. Decomposing plant materials including, deadwood and leaf litter are present together or in the vicinity with fine roots in the detritusphere and topsoil^24,27^. Closely related fungi even within the same genera have evolved differently, by specializing in these substrates^17^. While saprotroph fungi have evolved toward litter and deadwood materials with increasing/maintaining of plant cell wall degrading enzymes related genes (especially Carbohydrate-Active EnZymes (CAZymes)), many EM fungi displayed reduction of such genes, while maintaining and increasing symbiosis related genes such as sugar transporters, transposable elements (TEs) and small secreted proteins (SSPs)^17,28^. However, some EM fungi have retained substantial numbers of plant cell-wall degrading genes and have been found to have the ability to decompose plant organic matter^17,29,30^. Thus, deadwood and leaf litter can be considered as alternative substrates for such EM fungi, in life phases or compartments where they cannot establish ectomycorrhiza^6,11,12,31^. In forest ecosystem, it is also common that fine root patches of diverse trees colonize decomposing deadwood and leaf litter, from which they obtain nutrients via their EM fungal partners. When EM fungi can survive as saprotroph on decomposing plant materials (especially deadwood), they can increase their potential to find right host plants and to increase the chance of successful colonization^6^.

The plasticity of symbiotroph-saprotroph EM fungal lifestyles has been hypothesized for more than a decade^32^. A study also demonstrated that many saprotrophic fungi are able to colonize fine roots of ectomycorrhizal trees and form either incomplete or complete ectomycorrhizas, indicating the potential for such plasticity^29^. However, thus far there is no substantial evidence that demonstrates the efficient colonization of deadwood and its components by EM fungi and whether EM fungi-colonizing deadwood can change their lifestyles from saprotrophic to ectomycorrhizal programs. The dual role of EM fungi as a symbiotroph and a saprotroph therefore still under debate^33^.

In this study, *Piloderma croceum* associated with *Quercus robur* L. was used to demonstrate the plasticity of symbiotroph-saprotroph lifestyles of EM fungi. The saprotroph lifestyle can be demonstrated by the ability of *Piloderma croceum* to colonize and decompose dead organic matter from wood. The decomposition products (especially glucose) and nutrients released from dead organic matter have to be incorporated into the mycelium of the fungus. To demonstrate the switch from the saprotroph to the symbiotroph- lifestyle, *P. croceum* living in the saprotroph stage on dead wood, had to be able to colonize the living roots of *Q. robur* plants and exhibit a clear symbiotic apposition structure (i.e. Hartig net), as well as a benefits of EM formation, i.e. tree growth stimulation. Therefore, in this current work nine experiments were carried out to test whether (1) *P. croceum* can colonize a decomposing plant material, deadwood (bark, outer sapwood, inner sapwood, and the complete deadwood) of *Quercus robur* in the absence of living oak plants (experiments 1 to 4) and (2) such colonized decaying wood can serve as a propagule bank for forming new mycorrhizal symbiosis when faced with roots of living oaks (experiments 5 to 9). Specifically, we aimed to answer four questions. First, could *P. croceum* grow and persist on bark and wood substrates (experiment 1)? This would demonstrate the capacity to live or persist when not in symbiosis with roots (but not yet to establish a saprotrophic lifestyle). Second, what was the fungal enzymatic activity in wood colonized by *P. croceum* (experiment 2)? This would demonstrate that the fungus can release enzymes (as expected) that are capable of decomposing polymers of wood fibers, i.e., that it is truly saprotrophic. Third, was any wood carbon or related products, as measured via all-*E*-tetradeca-2, 4, 6, 8, 10, 12-hexaene-l,14-dioic acid (corticrocin) (experiment 3) and carbon isotope (^14^C) (experiment 4), detectable in vegetative tissue of the fungus? This would further demonstrate that the fungus is capable of decomposition and assimilating carbon and glucose from wood fibers and polymers, i.e., saprotrophic. Fourth, can deadwood be a source of dispersal for *P. croceum* to establish ectomycorrhizal roots (experiments 5 to 9)? This would show that wood can be a source of inoculants for the fungus, a key tenant of the research.

Based on plasticity of symbiotroph-saprotroph lifestyles of EM fungi, we hypothesized that *P. croceum* is able to grow and persist on bark and wood substrates^32^ (hypothesis 1). We hypothesized that based on its gene repertoire, *P. croceum* can secrete both hydrolytic (β-glucosidase) and oxidative enzymes (laccase and peroxidases) that are important for cellulose/hemicellulose and lignin degradation during an exclusive saprotrophic life phase^17,18^ (hypothesis 2). Furthermore, we expected that *P. croceum* would produce N-acetyl glucosaminidases and acid phosphatases to acquire N from chitin and organic P. Because of its ability to produce enzymes for C acquisition and lignin degradation/modification, we also hypothesized that the C released from deadwood could be transferred to and assimilated by *P. croceum* mycelium (hypothesis 3). We expected that *Q. robur* plants associated with *P. croceum* originating from deadwood would show the benefit of EM formation^34^ and their plasticity to outsource C with higher principal root, lateral root, and total root biomass, and stimulate plant growth with higher stem, shoot, and total plant biomass, compared with non-inoculated plants (hypothesis 4).

## Results and discussion

**Fig. 1:**
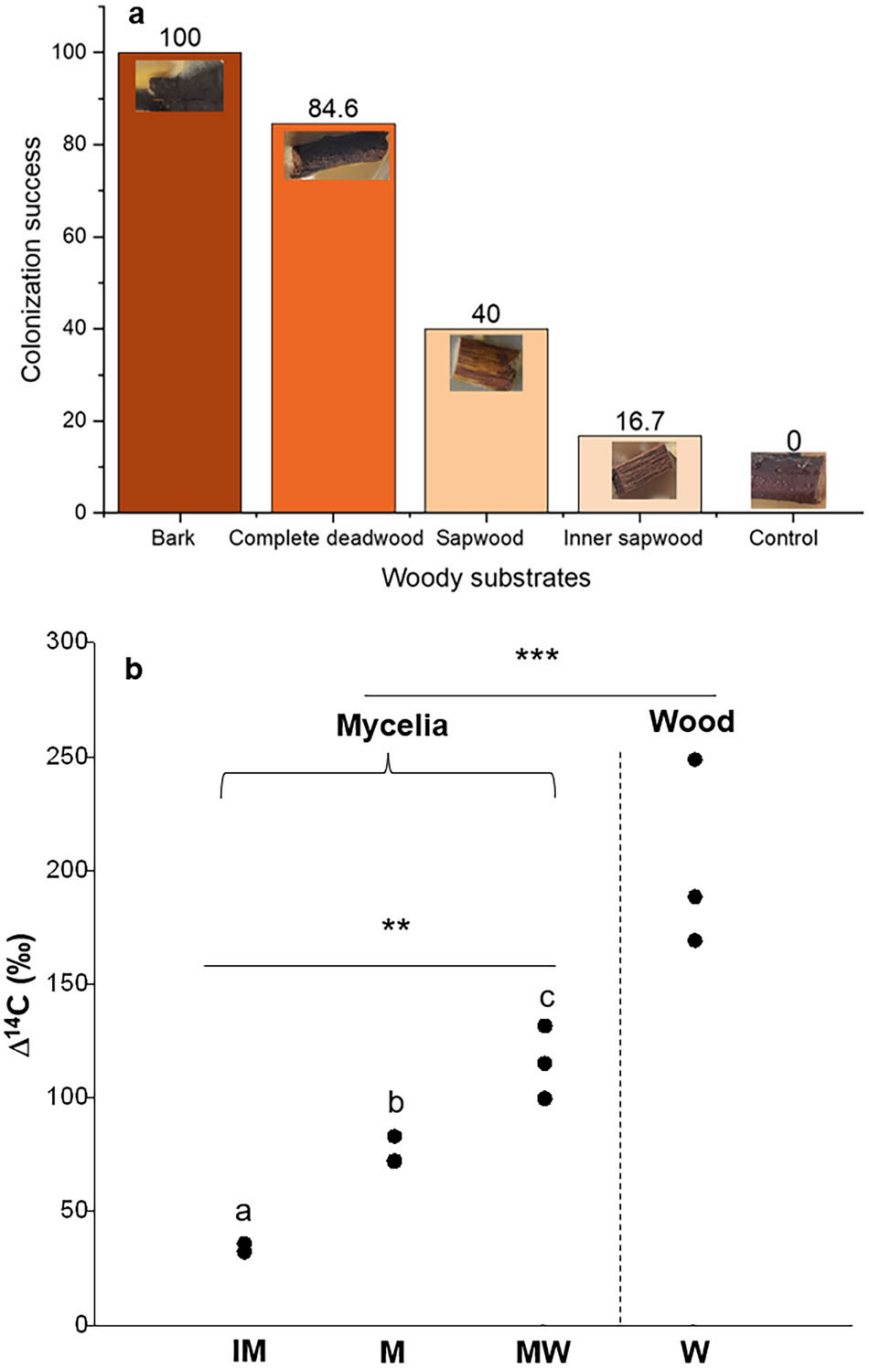
Saprophytic ability of *P. croceum*. Saprophytic ability **a** colonization success of different woody materials and **b** increase in Δ^14^C in mycelium (mean ± SE) of *P. croceum* that assimilated C from the introduced wood (IM = initial mycelium of *P. croceum*, M = 4-month mycelium of *P. croceum*, MW = 4-month mycelium of *P. croceum* growing near inner sapwood, and W = inner sapwood). Δ^14^C values of agar media are provided in Table S1. Different letters indicated significant differences of Δ^14^C (‰) among mycelial types according to one-way ANOVA with post hoc Tukey’s HSD test at *p* < 0.05 (*n* = 3). Differences of Δ^14^C (‰) between mycelium and wood were analyzed using a *t*-test at *p* < 0.05 (*n* = 3). ***p < 0.001, **p < 0.01.

**Fig. 2:**
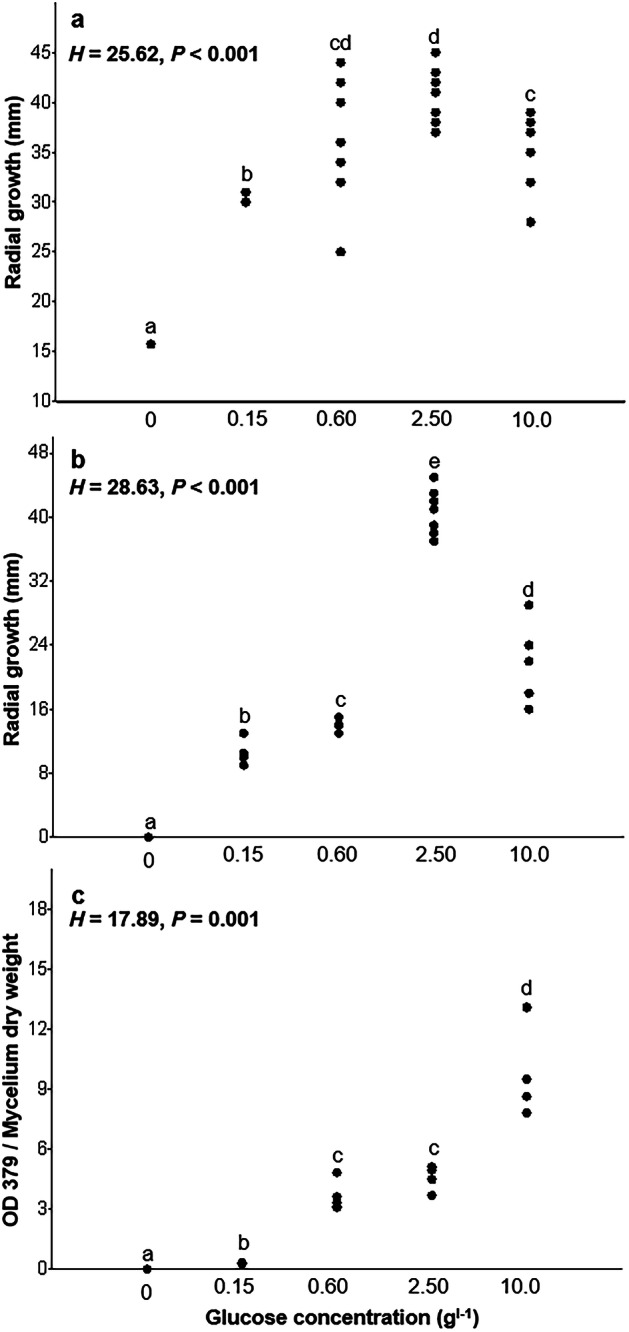
Effect of glucose on growth of *P. croceum* and corticrocin production after 9 weeks of cultivation on MMN1/10 with different available glucose concentrations (0.15, 0.6, 2.5, 10.0 g l^−1^). **a**, **b** Give the diameter of the total and yellow (mean ± SE), respectively. Different letters indicated significant differences of the diameter of the total and yellow colony among different available glucose concentrations according to Kruskal–Wallis test with post hoc Mann–Whitney pairwise test at *p* < 0.05 (*n* = 5–7). **c** Gives the ratio between optical density of corticrocin extracts (OD_379_) and colony dry weight. Different letters indicated significant differences of the ratio between OD_379_ and colony dry weight among different available glucose concentrations according to Kruskal–Wallis test with post hoc Mann–Whitney pairwise test at *p* < 0.05 (*n* = 4). The test statistic *H* and *p* values from Kruskal–Wallis test are shown in the upper left-hand corner of each figure.

*P. croceum* efficiently colonized deadwood of oaks in the experimental system without living oak, however, the rate of colonization after 4 months of incubation time varied depending on the substrate (100 in bark, 84.6 in complete wood, 40 in sapwood, and 16.7% in inner sapwood) (Fig. 1a and Supplementary Data 1, experiment 1). This finding answers our first question (hypothesis 1) and demonstrates the capacity of *P. croceum* to live or persist when not in symbiosis with roots of *Q. robur*. The earliest colonization of deadwood by *P. croceum* was observed after 2 weeks to 1 month in the bark and complete wood samples (Figs. 1 and S1). At this stage, white mycelium covered parts of the deadwood near the inoculated areas. After 1–2 months, the white mycelium became yellow and extended its development on deadwood (Figs. 1 and S1). After 4 months, the bark and complete wood were covered with massive amounts of *P. croceum* yellow mycelium, whereas the colonization on sapwood and inner sapwood was moderate. The yellow pigment synthesized by the mycelium of *P. croceum* has been identified as corticrocin, which is formed during colonization of mycorrhizal host roots^35^. We demonstrate here that the production of the yellow pigment, as indicated by the size of yellow colonies (*p* < 0.001) and optical density (OD) 379 (*p* < 0.001), was significantly influenced by the available glucose and growth of *P. croceum* in pure culture (Fig. 2a–c and Supplementary Data 2). Thus, corticrocin production in the mycelium of *P. croceum* colonizing deadwood in MMN 1/10 medium without any other soluble carbohydrate source, but with deadwood substrate, is a good indicator that *P. croceum* can partly decompose bark and sapwood to acquire organic carbon and glucose, which is consistent with our hypothesis 3. Substantial amount of glucose released from deadwood is required for corticrocin synthesis, thus *P. croceum* had to decompose deadwood over 1–2 months to reach such level, which also depended on the types of the deadwood materials. Our results show that on fresh bark fastest colonization rates are displayed by a yellow *P. croceum* mycelium that produces corticrocin. A recent study demonstrated that for temperate trees, the non-structural carbohydrates (NSC especially sugars and starch) are the most concentrated, in the outermost stem wood^36^. In contrast, the concentrations of NSC is lower, when the mycelium colonization is progressing deeper toward the pith^36^. We hypothesize that *P. croceum* takes advantage from the higher concentration of NSC at the outermost part of the stem (bark and vicinity area) to synthesize the corticrocin using the beta-glucosidase produced. Further studies are needed to confirm what supports *P. croceum* growth in the bark, whereby it is documented in literature that the front of mycelium colonies may recycle resources accumulated in their older part^37^.

Hydrolytic and oxidative enzyme activities in complete wood colonized by *P. croceum* were measured using microplate assays^38,39^. The results show that *P. croceum* produced significant amounts of hydrolytic enzymes on deadwood (*p* = 0.007–0.009), including β-glucosidase (588.4 ± 54.7 nmol h^−1^ g dry wood^−1^, mean ± SE), N-acetylglucosaminidase (717.2 ± 157.5, mean ± SE), and acid phosphatase (603.7 ± 95.1, mean ± SE), whereas the activities of oxidative enzymes, including laccase and peroxidase, were undetectable (Table 1). Thus, the glucose available for corticrocin formation was apparently related to β-glucosidase activity, which enables the hydrolysis of cellulose or hemicellulose from *Q. robur* bark or sapwood. The found activity of N-acetyl glucosaminidase and acid phosphatase demonstrated that *P. croceum* was also able to recycle N via chitin degradation from part of its own mycelium, and to acquire phosphorus from the wood substrate. The chitinase activity of *P. croceum* is directly related to earlier observations of *P. involutus*. Under C starvation, *P. involutus* uses autolysis with the associated chitinase activity to recycle N and C from its dead fungal mycelium^37^. The results from enzymatic assays demonstrate that *P. croceum* can release hydrolytic enzymes that are capable of decomposing wood fibers, i.e., to adopt a saprotrophic lifestyle in deadwood (hypothesis 2). The absence of oxidative enzymes, including laccase and peroxidase observed in this study, may reflect either a limited potential of *P. croceum* to produce lignin degrading/modifying enzymes^17^, or a potential not to produce these enzymes as long as more readily available resources are available. Production of oxidative enzymes important for lignin modification/degradation is more energy intensive than for hydrolytic enzymes, and also requires more resources, such as copper and manganese^40^. These metals are limited in our experimental setup, which contains only deadwood on diluted Modified Melin-Norkrans 1/10 medium.

**Table 1:** Hydrolytic and oxidative enzyme activities (mean ± SE, nmol h^−1^ g dry wood^−1^) on complete oak deadwood with and without (control) *P. croceum*

Enzyme	Degrading substrates	Control oak wood	Oak wood with *P. croceum*
Beta-glucosidase	Cellulose/hemicellulose	0.0 ± 0.0^a^	588.4 ± 54.7^b^
N-acetyl glucosaminidase	Chitin	1.4 ± 1.4^a^	717.2 ± 157.5^b^
Acid phosphatase	Organic phosphate	0.0 ± 0.0^a^	603.7 ± 95.1^b^
Laccase	Lignin	0.0 ± 0.0	0.0 ± 0.0
Peroxidase	Lignin	0.0 ± 0.0	0.0 ± 0.0

Different letters indicated significant differences of enzyme activities between the oak deadwood with and without (control) *P. croceum* according to Mann–Whitney *U*-test at *p* < 0.05 (*n* = 5).

After we introduced the mycelia on deadwood with higher ^14^C content than on agar, the mycelium ^14^C content significantly increased from the agar value and initial mycelia (*p* < 0.01), further demonstrating the capability of wood decomposition and assimilation of C from the wood (hypothesis 3) (Fig. 1b and Supplementary Data 1). Taking the common definition of fungal saprotrophs as “fungi that are able to live independently from dead materials and to degrade organic residues contained therein to obtain carbon”^41^, all the evidence from this series of experiments demonstrates the saprophytic capacity of *P. croceum* when grown on deadwood as a unique organic substrate. Deadwood can be considered to be more resistant compared to other forms of plant materials such as leaf and fine root litter, as it can persist many years in temperate forest ecosystems^12^. Obviously, the colonization success of *P. croceum* on deadwood warrants its long lasting survival apart from symbiotic association. Our finding that bark appears to be preferentially colonized and decomposed by *P*. *croceum* can be explained by the higher concentration of macronutrients and mineral elements (especially N, Mg, Fe) in bark of *Q. robur* as compared to sapwood^42^. Furthermore, the concentration of non-structural carbohydrates, including sugars and starch is also higher in the outermost part of the wood than the inner part of the wood^36^. Such non-structural carbohydrates, macronutrients and mineral elements are crucial for microbial enzyme production and activities^43,44^. Furthermore, bark of *Quercus* spp. can be considered as an important source of secondary metabolites which may impact growth of *P*. *croceum*^45^.

**Fig. 3:**
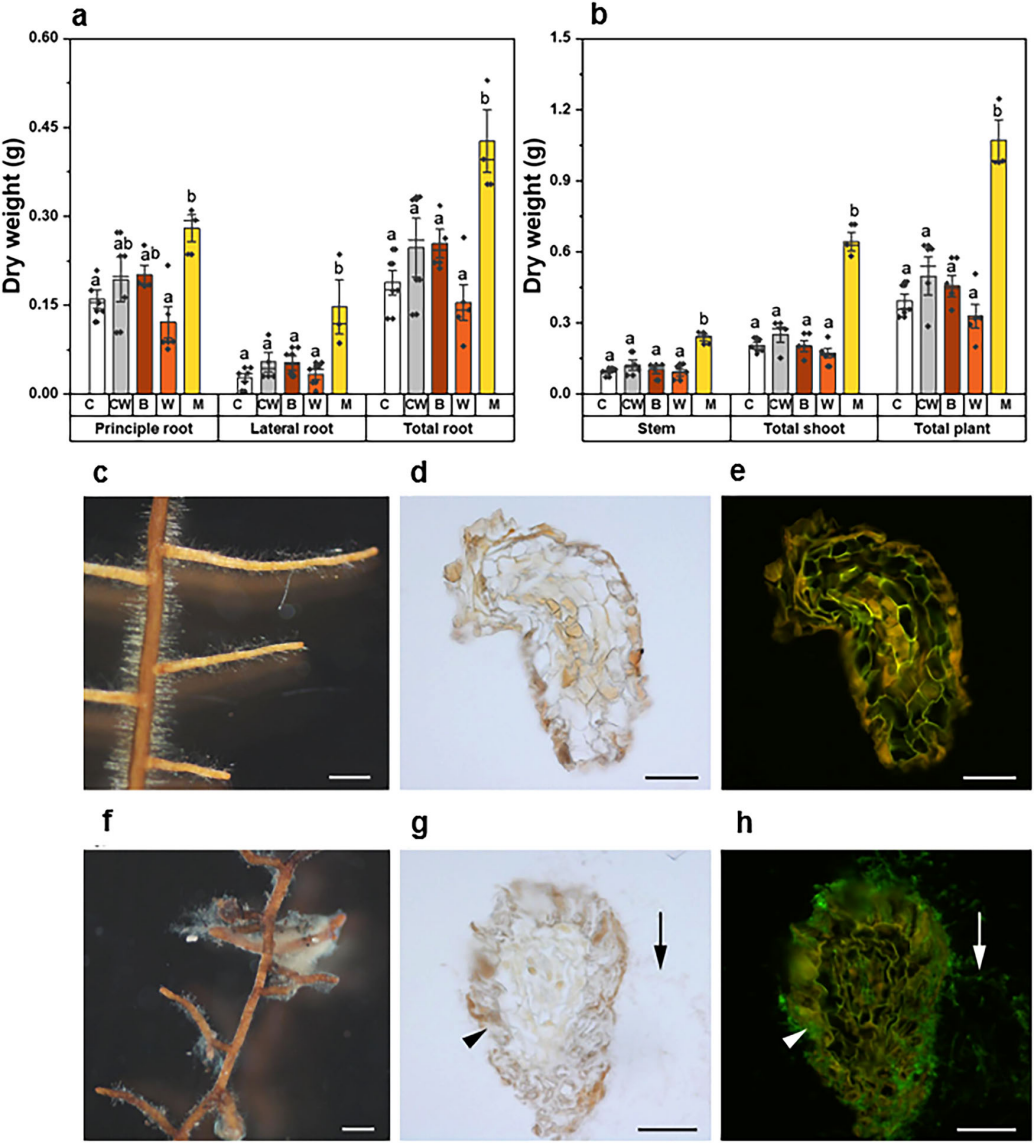
Role of deadwood as a source of propagule to form mycorrhizal symbiosis on roots of living *Quercus robur*. **a** Root dry weight (mean ± SE) and **b** plant growth parameters (stem, shoot, and total plant dry weight) (mean ± SE). C = control, CW = control with sterile complete oak wood, B = oak bark with *P. croceum*, W = oak deadwood with *P. croceum*, and M = oak and bark with *P. croceum* which formed yellow mycelium on oak root tips. Different letters indicated significant differences of the dry mass of roots and plant growth parameters according to one-way ANOVA with post-hoc Tukey’s HSD test at *p* < 0.05. **c**–**h** Microscopic analysis of oak roots in control and deadwood with *P. croceum* treatments. In contrast with the control, which did not show any fungal structures (**c**–**e**), mycelium of *P. croceum* was found to cover the root tips of oak (**f**) and the Hartig net was detectable in cross sections of paraffin-embedded root tips and stained with fluorescently labeled wheat germ agglutinin (**g**, **h**). Note the green fluorescence of outer mycelium (arrows) and Hartig net (arrowheads) in (**h**). Pictures were taken using a stereomicroscope (**c**, **f**), bright field (**d**, **g**), and fluorescence microscopy (**e**, **h**). Bars represent 500 µm in (**c**, **f**) and 50 µm in (**d**, **e**, **g**, **h**).

To evaluate the role of deadwood as a propagule bank to form mycorrhizal symbiosis on the roots of living *Q. robur*, we introduced colonized woody material from different types into the soil in the vicinity of the roots of living oaks under controlled conditions (Figs. 3 and S1 and Supplementary Data 3). Sterile oak wood without *P. croceum* was used as the control. *P. croceum* was detected in the rhizosphere of oak plants after 14 weeks in most inoculation treatments; however, only in the bark (one out of five replicates) and complete deadwood (two out of seven replicates) treatments was yellow mycelium of *P. croceum* visible on the oak roots (Supplementary Data 4 and Fig. S1). The oak plants with visible *P. croceum* mycelium on the roots had significantly higher dry biomass of principle root (*p* = 0.008), lateral roots (*p* = 0.003), total roots (*p* = 0.001), stem (*p* < 0.001), shoot (*p* < 0.001), and total plant (*p* < 0.001) compared to other oak plants, regardless of treatment (hypothesis 4) (Fig. 3a, b and Supplementary Data 3). We did not detect *P. croceum* in the control treatment, where the oak plant did not benefit from EM formation, but we detected *P. croceum* in the oak rhizosphere of all inoculation treatments using the Illumina sequencing approach. However, we detected this fungus frequently (relative abundance >50%) only in the treatments consisting of sterile soil with deadwood containing visible yellow mycelium of *P. croceum* (Supplementary Data 4). Microscopic observations of root tips surrounded by *P. croceum* mycelia showed the formation of a Hartig net (Fig. 3g, h), indicating a typical ectomycorrhizal symbiotic interaction. These results show that in presence of wood pieces colonized by saprotrophic mycelium of *P. croceum*, the roots of oak plants were not only superficially colonized by yellow mycelium, but also developed true EM symbiosis with Hartig net formation. Although noteworthy because *P. croceum* requires a delay and a certain carrying capacity of the plant to form fully differentiated EM with a Hartig net^46^, this result indicates that the inoculation efficiency provided from the pre-colonized deadwood can urge full EM differentiation.

Overall, we demonstrated the plasticity of the symbiotroph-saprotroph lifestyles of ectomycorrhizal *P. croceum* associated with *Q. robur*, which is in line with the hypotheses from the earlier studies^29,32^. The past field observations, based on fruiting bodies, were focused on the mycorrhizal stage and not on the saprophytic stage. However, molecular tools have allowed the detection of the dual lifestyle^17,18^. Importantly, we demonstrated the role of deadwood as a de novo ectomycorrhizal propagule bank. We provided evidence that *P. croceum* survives on oak deadwood for at least 2 years (Fig. S2). Thus, deadwood can influence forest health and productivity by acting as a substrate for a pool of organisms that can optionally be symbiotrophs or saprotrophs. Future studies, especially using transcriptomics are needed to gain a better understanding of mechanisms behind the plasticity of the symbiotroph-saprotroph lifestyles of ectomycorrhizal fungi.

## Materials and methods

### *P. croceum* as saprotroph on *Q. robur* deadwood

*Experiment 1*: Could *P. croceum* grow and persist on bark and deadwood substrates?—Colonization success experiment (experimental design based on 74 deadwood materials).

This experiment was used to test the hypothesis 1. *P. croceum* J. Erikss. and Hjortst isolate DSMZ 4824; ATCC MYA-4870 was maintained under dark conditions at 20 °C on Modified Melin-Norkrans C (MMNC) agar medium with sugar and subsequently transferred to Modified Melin-Norkrans (MMN) 1/10 medium (no sugar or other carbohydrate sources) for 1 month in a Petri dish (94 mm)^47,48^. After 3.5 months, different woody substrates (including bark with nine replicates), outer sapwood with 15 replicates, and complete deadwood with 26 replicates) were obtained from 2 cm branches of *Q. robur* L., obtained from older clonal field trees DF159^47^ were autoclaved thrice and placed on the top of the growing colony. Additionally, we included inner sapwood (cutting the tree rings between 1955 and 1963 and autoclaving three times, 24 replicates) for this colonization success experiment. These inner sapwood samples contained a significant amount of ^14^C compared with recently collected normal oak wood. Colonization of *P. croceum* was observed over 4 months. Contamination was not detected in the control group. Percent colonization was calculated for each woody substrate and used as the indicator for colonization success.

*Experiment 2*: What was the enzymatic activity of wood colonized by *P. croceum*?—Hydrolytic and oxidative enzymes in deadwood. This experiment was used to test the hypothesis 2.

Sterile complete deadwood obtained from oak branches of DF159 trees was placed on MMN 1/10 agar with and without *P. croceum* (control) with the same setting as experiment one for 4 months (maintained under dark conditions at 20 °C). The wood samples were homogenized and ground into powder. Hydrolytic enzyme analyses were performed for β-glucosidase (EC 3.2.1.21), N-acetylglucosaminidase (EC 3.1.6.1), and phosphatase (EC 3.1.3.2), using 4-methylumbelliferone (MUB) as a substrate, as described in previous publications^23,38,39^. Oxidative enzymes (phenol oxidase (EC 1.10.3.2) and general peroxidase (EC 1.11.1.7) were analyzed using 3, 3′, 5, 5′-tetramethylbenzidine (TMB) substrate, as described in our previous study^39^. The experiment was conducted with 5 replicates and differences of each enzyme activity between control wood and wood with *P. croceum* were analyzed using a Mann-Whitney U-test.

*Experiment 3*: Corticrocin production and its association with available glucose—We varied the concentration of glucose in the MMN 1/10 media (0.0, 0.15, 0.6, 2.5, and 10.0 g/L) used to cultivate *P. croceum* J. Erikss. and Hjortst isolate DSMZ 4824; ATCC MYA-4870.

The culture plates were incubated at 20 °C for 9 weeks in dark. The dry weight and diameters of the total and yellow part of the colonies were determined. Corticrocin production was measured at the optical density OD 379. The experiment consisted of five to seven replicates for dry weight and diameter and four replicates for destructive OD measurements. Data were tested for normality and equality of group variance. Significant differences of the diameter of the total and yellow colony among different available glucose concentrations were tested using Kruskal–Wallis test incorporated with Mann–Whitney pairwise comparisons at *p* < 0.05. This experiment was used to test the hypothesis 3 that *P. croceum* can release enzymes that are capable of decomposing wood fibers and polymers into different C forms, including glucose. Thus, glucose is important for corticrocin production and strongly linked with corticrocin concentration in mycelium of *P. croceum*.

*Experiment 4*: Was any wood carbon, as measured via isotopes, detectable in vegetative tissue of the fungus?—Carbon released from deadwood, transported, and assimilated to *P. croceum* mycelium.

To demonstrate the uptake of wood carbon by mycelia, we used wood with higher ^14^C content than the MMNC and MMN 1/10 agar media (Δ^14^C values of agar media are provided in Table S1). This experiment was used to test the hypothesis 3. We observed wood that grew four decades ago from slices of *Q. robur* trunks. At this time, the Δ^14^C in atmospheric CO_2_ was ~150‰ higher than that in agar medium^49^. The high atmospheric Δ^14^C signature is imprinted in the wood produced in those years of the atmosphere. We initially cultured *P. croceum* mycelium on MMNC agar medium (*n* = 3). Then we used the initial mycelium to inoculate cultures on C-poor MMN 1/10 agar and maintained them under dark conditions at 20 °C, one set with wood pieces, and the second control set without wood. The samples were analyzed by the Keck Carbon Cycle AMS facility at the University of California, Irvine, and in the radiocarbon laboratory in Jena, Germany. Significant differences of Δ^14^C (‰) among mycelial types were analyzed according to one-way ANOVA at *p* < 0.05. Differences of Δ^14^C (‰) between mycelia and wood were analyzed using a *t*-test. Data were tested for normality and equality of group variance.

### *P. croceum* colonized oak deadwood changes its lifestyle from saprotroph to ectomycorrhizal in *Q. robur*

*Experiment 5*: Can deadwood be a source of dispersal by *P. croceum* for establishing ectomycorrhizal roots?—Deadwood inoculation and evaluation of symbiotic success.

Woody materials from Experiment 1 were used in this experiment. Because of the lower colonization success of sapwood and inner sapwood, we did not include them in this experiment. This experiment consisted of four treatments with five to seven replicates (sterile soil without deadwood (five replicates), sterile soil with sterile complete deadwood, bark, and sapwood (five replicates), sterile soil with bark containing *P. croceum* (five replicates), and sterile soil with dead wood containing *P. croceum* (seven replicates) (Fig S1). The oak DF159 micro-cuttings^47^ were maintained for 14 weeks at 23 °C before evaluation of the symbiosis success. The root system grew in the Petri dish under dark conditions and the shoot under a 16:8 h day: night photoperiod (photosynthetic photon flux density of 180 µmol m^−2^ s^−1^). Symbiosis success was indicated by visible yellow mycelium on the roots of oak micro-cuttings. Furthermore, we confirmed the success of symbiosis by Illumina sequencing of the rhizosphere of oak micro-cuttings with and without yellow mycelium (Experiment 7) and by microscopic observation (Experiment 8).

*Experiment 6*: Ectomycorrhizal effects—Determination of ectomycorrhizal effects.

This experiment was used to test the hypothesis 4. We evaluated the ectomycorrhizal effects on all micro-cuttings from Experiment 5. Based on our previous publication, *P. croceum* is known to positively impact the dry mass of roots (principal roots, lateral roots, and total roots) and plant growth parameters (dry mass of stem, shoot, and total plant)^34^. Therefore, these parameters were evaluated. Plant dry weight was determined by oven-dry at 105 °C for 24 h or until constant weight. Significant differences of the dry mass of roots and plant growth parameters were analyzed using one-way ANOVA at *p* < 0.05. Data were tested for normality and equality of group variance.

*Experiment 7*: The existence of *P. croceum*—Molecular detection of *P. croceum* in the rhizosphere of *Q. robur* DF159.

The rhizospheres of oak micro-cuttings from the same treatments were pooled, sterile soil without deadwood, sterile soil with sterile complete deadwood, sterile soil with bark containing *P. croceum* (without yellow mycelium), sterile soil with bark containing *P. croceum* (with yellow mycelium), sterile soil with deadwood containing *P. croceum* (without yellow mycelium), and sterile soil with deadwood containing *P. croceum* (with yellow mycelium). DNA was extracted from 250 mg of the pooled rhizosphere soil and soil samples using DNeasy PowerSoil Kit (Qiagen, Hilden, Germany) with aid of a Precellys 24 tissue homogenizer (Bertin Instruments, Montigny-le-Bretonneux, France). Illumina sequencing and bioinformatics analyses were performed according to our published protocol^50^. Briefly, fungal amplicon libraries were constructed using the fungal primer pair fITS7 (5′-GTGARTCATCGAATCTTTG-3′) and ITS4 primer (5′-TCCTCCGCTTATTGATATGC-3′)^51^. Amplifications were performed using 20-µL reaction volumes with 5 × HOT FIRE Pol Blend Master Mix (Solis BioDyne, Tartu, Estonia). The products from three technical replicates were then pooled in equimolar concentrations. Paired-end sequencing (2 × 300 bp) was performed on the pooled PCR products using a MiSeq Reagent kit v3 on an Illumina MiSeq system (Illumina Inc., San Diego, CA, United States) at the Department of Soil Ecology, Helmholtz Centre for Environmental Research, Germany. Paired-end sequences were quality-trimmed, filtered for chimeras, and merged using the DADA2 package^52^. High-quality reads were clustered into amplicon sequence variants (ASVs). Fungal ASVs were classified against the UNITE v7.2 database^53^. Sequencing was performed carefully by pooling negative control of all PCR runs and used as sequencing control. Raw sequence data were deposited in the Sequence Read Archive (SRA) operated by the National Center for Biotechnology Information (NCBI) under BioProject accession number: PRJNA930471.

*Experiment 8*: *P. croceum* ectomycorrhizal structure—Observation of *P*. *croceum* structure in roots of *Q. robur*.

Tips from oak roots grown without *P. croceum* (control) and on deadwood with *P. croceum* (sample T3E2.2 005) were fixed with 4% (w/v) paraformaldehyde/ 0.1% (v/v) TritonX-100 in phosphate-buffered saline (PBS). Micrographs were obtained using a STEMI 2000c stereomicroscope (Zeiss GmbH, Jena, Germany) equipped with an AxioCam Mrc5 CCD camera (Zeiss). Then, the root tips were dehydrated using a graded series of ethanol and embedded in Paraplast (Sigma-Aldrich). Cross-sections of Paraplast-embedded material (5 µm thick) were mounted on poly *L*-lysine-coated slides, deparaffinized, and rehydrated. The sections were stained with 25 µg ml^−1^ wheat germ agglutinin conjugated with Alexa Fluor488 (Invitrogen) in PBS at room temperature for 20 min. After washing in PBS, micrographs were taken by bright-field and fluorescence (excitation BP 500/20, emission LP 515) microscopy using an AxioImager equipped with an AxioCam Mrc5 CCD camera (Zeiss).

*Experiment 9*: Can deadwood be a source of dispersal by *P. croceum* for establishing ectomycorrhizal roots?—Deadwood as an EM fungal Propagule Bank.

*P. croceum* was cultivated on MMN 1/10 medium (no sugar or other carbohydrate sources) for 1 month in a Petri-dish (94 mm). After 3.5 months, the complete deadwood (three replicates) was obtained from 2 cm branches of *Q. robur* L. obtained from clonal trees DF159^31^, which were autoclaved thrice and placed on the top of the growing colony. *P. croceum* was grown on MMN 1/10 medium and oak deadwood for 1 and 2 years. For the control treatment, sterile oak wood was placed on an MMN 1/10 medium. The colonies of *P. croceum* on MMNC agar, colonies of *P. croceum* on MMN 1/10 agar, and oak deadwood with and without *P. croceum* were placed on MMNC agar medium. Fungal growth and development of yellow mycelium of *P. croceum* were recorded after 2 weeks.

### Statistics and reproducibility

Differences of enzyme activities between the oak deadwood with and without (control) *P. croceum* were analyzed using to Mann–Whitney *U*-test at *p* < 0.05 (*n* = 5). Differences of Δ^14^C (‰) among mycelial types were analyzed using one-way ANOVA with post-hoc Tukey’s HSD (Honestly Significant Difference) test at *p* < 0.05 (*n* = 3). Differences of Δ^14^C (‰) between mycelium and wood were analyzed using a *t*-test at *p* < 0.05 (*n* = 3). Differences of the diameter of the total and yellow colony among different available glucose concentrations were analyzed using Kruskal-Wallis test with post hoc Mann–Whitney pairwise test at *p* < 0.05 (*n* = 5–7). Differences of the ratio between OD_379_ and colony dry weight among different available glucose concentrations were analyzed using Kruskal–Wallis test with post hoc Mann–Whitney pairwise test at *p* < 0.05 (*n* = 4). Differences of the dry mass of roots and plant growth parameters were analyzed using one-way ANOVA with post hoc Tukey’s HSD test at *p* < 0.05 (*n* = 3–5). For all parametric tests (one-way ANOVA and t-test), all datasets were tested for normality and equality of group variance. All statistical analyses were performed using the PAST program (version 2.17c)^54^.

### Reporting summary

Further information on research design is available in the Nature Portfolio Reporting Summary linked to this article.

## Supplementary information

Supplementary information

Description of Additional Supplementary Files

Supplementary Data 1-4

Reporting summary

## Data Availability

Raw sequences were deposited in the Sequence Read Archive (SRA) operated by the National Center for Biotechnology Information (NCBI) under BioProject accession number: PRJNA930471. Experimental design, evidence shows that *P. croceum* survives on oak deadwood for at least 2 years and ^14^C data for the agar media used in this study can be found in Supplementary information. Source data underlying graphs in the study can be found in Supplementary Data 1–3.

## References

[1] Anderson, I. C. & Cairney, J. W. G. Ectomycorrhizal fungi: exploring the mycelial frontier. *FEMS Microbiol. Rev.* **31**, 388–406 (2007). 17466031 10.1111/j.1574-6976.2007.00073.x

[2] Allen, M. F. & Kitajima, K. Net primary production of ectomycorrhizas in a California forest. *Fungal Ecol.* **10**, 81–90 (2014).

[3] Okada, K. H. & Matsuda, Y. Soil spore bank communities of ectomycorrhizal fungi in *Pseudotsuga japonica* forests and neighboring plantations. *Mycorrhiza* **32**, 83–93 (2022). 34989868 10.1007/s00572-021-01065-y

[4] Glassman, S. I., Levine, C. R., DiRocco, A. M., Battles, J. J. & Bruns, T. D. Ectomycorrhizal fungal spore bank recovery after a severe forest fire: some like it hot. * ISME J.* **10**, 1228–1239 (2016). 26473720 10.1038/ismej.2015.182 PMC5029211

[5] Glassman, S. I. et al. A continental view of pine-associated ectomycorrhizal fungal spore banks: a quiescent functional guild with a strong biogeographic pattern. *New Phytol*. **205**, 1619–1631 (2015). 25557275 10.1111/nph.13240

[6] Purahong, W. et al. Characterization of unexplored deadwood mycobiome in highly diverse subtropical forests using culture-independent molecular technique. *Front. Microbiol*. **8**, 574 (2017). 10.3389/fmicb.2017.00574 PMC5395659 28469600

[7] Ottosson, E. et al. Diverse ecological roles within fungal communities in decomposing logs of *Picea abies*. *FEMS Microbiol. Ecol.* **91**, fiv012 (2015). 25764460 10.1093/femsec/fiv012

[8] Purahong, W. et al. Increasing N deposition impacts neither diversity nor functions of deadwood-inhabiting fungal communities, but adaptation and functional redundancy ensure ecosystem function. *Environ. Microbiol.* **20**, 1693–1710 (2018). 29473288 10.1111/1462-2920.14081

[9] Hibbett, D. S., Gilbert, L. B. & Donoghue, M. J. Evolutionary instability of ectomycorrhizal symbioses in basidiomycetes. *Nature* **407**, 506–508 (2000). 11029000 10.1038/35035065

[10] Tedersoo, L., May, T. W. & Smith, M. E. Ectomycorrhizal lifestyle in fungi: global diversity, distribution, and evolution of phylogenetic lineages. *Mycorrhiza* **20**, 217–263 (2010). 20191371 10.1007/s00572-009-0274-x

[11] Rajala, T., Peltoniemi, M., Pennanen, T. & Mäkipää, R. Fungal community dynamics in relation to substrate quality of decaying Norway spruce (*Picea abies* [L.] Karst.) logs in boreal forests. *FEMS Microbiol. Ecol.* **81**, 494–505 (2012). 22458543 10.1111/j.1574-6941.2012.01376.x

[12] Purahong, W., Wubet, T., Krüger, D. & Buscot, F. Molecular evidence strongly supports deadwood-inhabiting fungi exhibiting unexpected tree species preferences in temperate forests. * ISME J.* **12**, 289–295 (2018). 29087376 10.1038/ismej.2017.177 PMC5739023

[13] Taylor, A. F. S., Martin, F. & Read, D. J. Fungal diversity in ectomycorrhizal communities of Norway Spruce [*Picea abies* (L.) Karst.] and Beech (*Fagus sylvatica* L.) along North-South transects in Europe. in *Carbon and Nitrogen Cycling in European Forest Ecosystems* (ed. Schulze, E.-D.) 343–365 (Springe, 2000).

[14] Mahmood, S., Finlay, R. D., Erland, S. & Wallander, H. Solubilisation and colonisation of wood ash by ectomycorrhizal fungi isolated from a wood ash fertilised spruce forest. *FEMS Microbiol. Ecol.* **35**, 151–161 (2001). 11295454 10.1111/j.1574-6941.2001.tb00799.x

[15] Kohler, A. et al. Convergent losses of decay mechanisms and rapid turnover of symbiosis genes in mycorrhizal mutualists. *Nat. Genet.* **47**, 410–415 (2015). 25706625 10.1038/ng.3223

[16] Chen, D. M., Bastias, B. A., Taylor, A. F. S. & Cairney, J. W. G. Identification of laccase-like genes in ectomycorrhizal basidiomycetes and transcriptional regulation by nitrogen in *Piloderma byssinum*. *New Phytol.* **157**, 547–554 (2003). 33873413 10.1046/j.1469-8137.2003.00687.x

[17] Miyauchi, S. et al. Large-scale genome sequencing of mycorrhizal fungi provides insights into the early evolution of symbiotic traits. *Nat. Commun.* **11**, 5125 (2020). 33046698 10.1038/s41467-020-18795-w PMC7550596

[18] Pellitier, P. T. & Zak, D. R. Ectomycorrhizal fungi and the enzymatic liberation of nitrogen from soil organic matter: why evolutionary history matters. *New Phytol.* **217**, 68–73 (2018). 29193221 10.1111/nph.14598

[19] Shah, F. et al. Ectomycorrhizal fungi decompose soil organic matter using oxidative mechanisms adapted from saprotrophic ancestors. *New Phytol.* **209**, 1705–1719 (2016). 26527297 10.1111/nph.13722 PMC5061094

[20] Bödeker, I. T. M. et al. Ectomycorrhizal *Cortinarius* species participate in enzymatic oxidation of humus in northern forest ecosystems. *New Phytol.* **203**, 245–256 (2014). 24725281 10.1111/nph.12791

[21] Rineau, F. et al. Carbon availability triggers the decomposition of plant litter and assimilation of nitrogen by an ectomycorrhizal fungus. *ISME J.* **7**, 2010–2022 (2013). 23788332 10.1038/ismej.2013.91 PMC3965319

[22] Santos, C. A. et al. An engineered GH1 β-glucosidase displays enhanced glucose tolerance and increased sugar release from lignocellulosic materials. *Sci. Rep.* **9**, 4903 (2019). 30894609 10.1038/s41598-019-41300-3 PMC6426972

[23] German, D. P. et al. Optimization of hydrolytic and oxidative enzyme methods for ecosystem studies. *Soil Biol. Biochem.* **43**, 1387–1397 (2011).

[24] Olchowik, J. et al. Effect of deadwood on ectomycorrhizal colonisation of old-growth oak forests. *Forests* **10**, 480 (2019).

[25] Purahong, W. et al. Potential links between wood-inhabiting and soil fungal communities: Evidence from high-throughput sequencing. *MicrobiologyOpen* **8**, e00856 (2019). 31134764 10.1002/mbo3.856 PMC6741142

[26] Lewis, J. D. Mycorrhizal fungi, evolution and diversification of. in *Encyclopedia of Evolutionary Biology* (ed. Kliman, R. M.) 94–99 (Academic Press, 2016).

[27] Lang, A. K., Jevon, F. V., Vietorisz, C. R., Ayres, M. P. & Hatala Matthes, J. Fine roots and mycorrhizal fungi accelerate leaf litter decomposition in a northern hardwood forest regardless of dominant tree mycorrhizal associations. *New Phytol.* **230**, 316–326 (2021). 33341954 10.1111/nph.17155

[28] Khan, F. K., Sánchez-García, M., Johannesson, H. & Ryberg, M. High rate of gene family evolution in proximity to the origin of ectomycorrhizal symbiosis in *Inocybaceae*. *New Phytol.* **244**, 219–234 (2024). 39113397 10.1111/nph.20007

[29] Smith, G. R., Finlay, R. D., Stenlid, J., Vasaitis, R. & Menkis, A. Growing evidence for facultative biotrophy in saprotrophic fungi: data from microcosm tests with 201 species of wood-decay basidiomycetes. *New Phytol.* **215**, 747–755 (2017). 28382741 10.1111/nph.14551

[30] Rúa, M. A. et al. Ectomycorrhizal fungal communities and enzymatic activities vary across an ecotone between a forest and field. *J. Fungi* **1**, 185 (2015). 10.3390/jof1020185 PMC5753110 29376908

[31] Tanunchai, B. et al. Tree mycorrhizal type regulates leaf and needle microbial communities, affects microbial assembly and co-occurrence network patterns, and influences litter decomposition rates in temperate forest. *Front. Plant Sci.* **14**, 1239600 (2023). 38094000 10.3389/fpls.2023.1239600 PMC10716483

[32] Koide, R. T., Sharda, J. N., Herr, J. R. & Malcolm, G. M. Ectomycorrhizal fungi and the biotrophy–saprotrophy continuum. *New Phytol.* **178**, 230–233 (2008). 18312537 10.1111/j.1469-8137.2008.02401.x

[33] Lindahl, B. D. & Tunlid, A. Ectomycorrhizal fungi—potential organic matter decomposers, yet not saprotrophs. *New Phytol.* **205**, 1443–1447 (2015). 25524234 10.1111/nph.13201

[34] Herrmann, S. et al. Endogenous rhythmic growth in oak trees is regulated by internal clocks rather than resource availability. *J. Exp. Bot.* **66**, 7113–7127 (2015). 26320242 10.1093/jxb/erv408 PMC4765786

[35] Schreiner, T., Hildebrandt, U., Bothe, H. & Marner, F.-J. Chemical and Biological characterization of corticrocin, a yellow pigment formed by the ectomycorrhizal fungus *Piloderma croceum*. *Z. für Naturforsch. C.* **53**, 4–8 (1998).

[36] Furze, M. E. et al. Seasonal fluctuation of nonstructural carbohydrates reveals the metabolic availability of stemwood reserves in temperate trees with contrasting wood anatomy. *Tree Physiol.* **40**, 1355–1365 (2020). 32578851 10.1093/treephys/tpaa080

[37] Akroume, E. et al. First evidences that the ectomycorrhizal fungus *Paxillus involutus* mobilizes nitrogen and carbon from saprotrophic fungus necromass. *Environ. Microbiol.* **21**, 197–208 (2019). 30307107 10.1111/1462-2920.14440

[38] Sinsabaugh, R. L. et al. Soil microbial activity in a Liquidambar plantation unresponsive to CO2-driven increases in primary production. *Appl. Soil Ecol.* **24**, 263–271 (2003).

[39] Purahong, W. et al. Tree species, tree genotypes and tree genotypic diversity levels affect microbe-mediated soil ecosystem functions in a subtropical forest. *Sci. Rep.* **6**, 36672 (2016). 27857198 10.1038/srep36672 PMC5114573

[40] Wong, D. W. S. Structure and action mechanism of ligninolytic enzymes. *Appl. Biochem. Biotechnol.* **157**, 174–209 (2009). 18581264 10.1007/s12010-008-8279-z

[41] Müller, K. et al. Saprotrophic and ectomycorrhizal fungi contribute differentially to organic P mobilization in beech-dominated forest ecosystems. *Front. For. Glob. Change* **3** (2020).

[42] Krutul, D. et al. Influence of urban environment originated heavy metal pollution on the extractives and mineral substances content in bark and wood of oak (*Queecus robur* L.). *Wood Res.* **59**, 177–190 (2014).

[43] Prescott, L., Harley, J. & Klein, D. *Microbiology* (McGraw-Hill Higher Education, 1999).

[44] Purahong, W. et al. Effects of forest management practices in temperate beech forests on bacterial and fungal communities involved in leaf litter degradation. *Microb. Ecol.* **69**, 905–913 (2015). 25749938 10.1007/s00248-015-0585-8

[45] Sirgedaitė-Šėžienė, V., Čėsnienė, I., Leleikaitė, G., Baliuckas, V. & Vaitiekūnaitė, D. Phenolic and antioxidant compound accumulation of *Quercus robur* bark diverges based on tree genotype, phenology and extraction method. *Life* **13**, 710 (2023). 36983864 10.3390/life13030710 PMC10051228

[46] Herrmann, S., Oelmüller, R. & Buscot, F. Manipulation of the onset of ectomycorrhiza formation by indole-3-acetic acid, activated charcoal or relative humidity in the association between oak microcuttings and *Piloderma croceum*: influence on plant development and photosynthesis. *J. Plant Physiol.* **161**, 509–517 (2004). 15202707 10.1078/0176-1617-01208

[47] Herrmann, S., Munch, J.-C. & Buscot, F. A gnotobiotic culture system with oak microcuttings to study specific effects of mycobionts on plant morphology before, and in the early phase of, ectomycorrhiza formation by *Paxillus involutus* and *Piloderma croceum*. *New Phytol.* **138**, 203–212 (1998). 33863094 10.1046/j.1469-8137.1998.00105.x

[48] Schubert, R. et al. Quantitative detection of agar-cultivated and rhizotron-grown *Piloderma croceum* Erikss. & Hjortst. by ITS1-based fluorescent PCR. *Mycorrhiza* **13**, 159–165 (2003). 12836084 10.1007/s00572-002-0212-7

[49] Trumbore, S. E., Sierra, C. A. & Hicks Pries, C. E. Radiocarbon nomenclature, theory, models, and interpretation: measuring age, determining cycling rates, and tracing source pools. in *Radiocarbon and Climate Change: Mechanisms, Applications and Laboratory Techniques* (eds Schuur, E. A. G., Druffel, E. & Trumbore, S. E.) 45–82 (Springer International Publishing, 2016).

[50] Tanunchai, B. et al. Analysis of microbial populations in plastic–soil systems after exposure to high poly(butylene succinate-co-adipate) load using high-resolution molecular technique. *Environ. Sci. Eur.* **33**, 105 (2021).

[51] Ihrmark, K. et al. New primers to amplify the fungal ITS2 region—evaluation by 454-sequencing of artificial and natural communities. *FEMS Microbiol. Ecol.* **82**, 666–677 (2012). 22738186 10.1111/j.1574-6941.2012.01437.x

[52] Callahan, B. J. et al. DADA2: high-resolution sample inference from Illumina amplicon data. *Nat. Methods* **13**, 581–583 (2016). 27214047 10.1038/nmeth.3869 PMC4927377

[53] Nilsson, R. H. et al. The UNITE database for molecular identification of fungi: handling dark taxa and parallel taxonomic classifications. *Nucleic Acids Res.* **47**, D259–D264 (2019). 30371820 10.1093/nar/gky1022 PMC6324048

[54] Hammer, Ø, Harper, D. A. T. & Ryan, P. D. PAST: paleontological statistics software package for education and data analysis. *Palaeontol. Electron.* **4**, 9 (2001).

