# Supplementary material for: Plasticity of symbiotroph-saprotroph lifestyles of Piloderma croceum associated with Quercus robur L
Source: Commun Biol. 2025 Sep 16;8:1344. doi: 10.1038/s42003-025-08762-w (PMC12441137; doi:10.1038/s42003-025-08762-w)
Supplement: Supplementary file 1 — Supplementary information [file 42003_2025_8762_MOESM1_ESM.pdf]

Supplementary information

Title: Plasticity of symbiotroph-saprotroph lifestyles of *Piloderma croceum* associated with *Quercus robur* L

Witoon Purahong[1*¶], Benjawan Tanunchai[1,2¶], Li Ji[1,3], Hagen Stellmach[4], Boaz Hilman[5], Ernst-Detlef Schulze[5], Bettina Hause[4], Mika Tarkka[1], François Buscot[1,6], Sylvie Herrmann[1*]

[1]UFZ-Helmholtz Centre for Environmental Research, Department of Soil Ecology, Theodor-Lieser-Str. 4, 06120 Halle (Saale), Germany

[2]Bayreuth Center of Ecology and Environmental Research (BayCEER), University of Bayreuth, Bayreuth, Germany

[3]Key Laboratory of Sustainable Forest Ecosystem Management-Ministry of Education, School of Forestry, Northeast Forestry University, 150040 Harbin, P.R. China

[4]Leibniz-Institut für Pflanzenbiochemie, Weinberg 3, 06120 Halle, Germany

[5]Max Planck Institute for Biogeochemistry, Biogeochemical Processes Department, Hans-Knöll-Str. 10, 07745 Jena, Germany.

[6]German Centre for Integrative Biodiversity Research (iDiv), Halle-Jena-Leipzig, Deutscher Platz 5e, 04103 Leipzig, Germany

*** Correspondence:** Corresponding Authors witoon.purahong@ufz.de, sylvie.herrmann@ufz.de

[¶] These authors contribute equally.

Figure S1 Experimental design on role of deadwood as a propagule bank to form mycorrhizal symbiosis on roots of living *Q. robur*. Deadwood inoculation and evaluation of symbiosis success experiment consists of control oak wood without *P. croceum* (complete wood with bark and sapwood), oak bark with *P. croceum* and complete wood with *P. croceum* (successful colonization of *P. croceum* on oak roots are shown in right panel indicate with green circle). MMNC = Modified Melin-Norkrans C agar medium (with sugar (glucose) and other carbohydrate sources) and MMN1/10 = Modified Melin-Norkrans (without sugar or other carbohydrate sources).

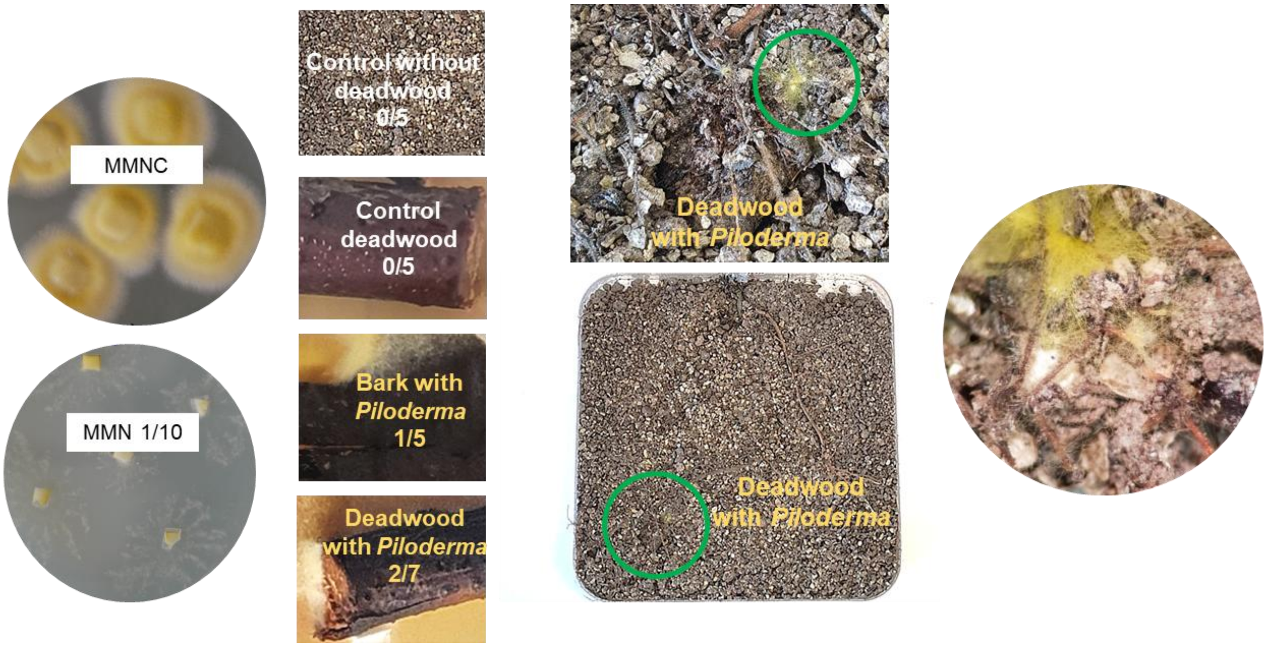

Figure S2 Evidence shows that *P. croceum* survives on oak deadwood for at least two years. MMNC = Modified Melin-Norkrans C agar medium (with sugar (glucose) and other carbohydrate sources) and MMN1/10 = Modified Melin-Norkrans (without sugar or other carbohydrate sources)

One year

Initial mycelium in MMNC agar medium = No *P. croceum* colony detected.

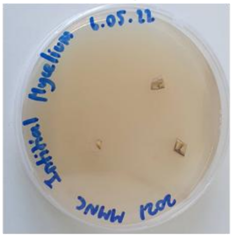

Mycelium in MMN 1/10 agar medium = No *P. croceum* colony detected.

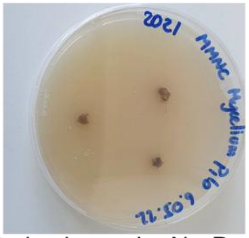

Sterile control oak wood = No *P. croceum* colony/active mycelium detected.

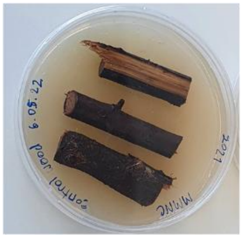

P. croceum inoculated oak wood = No *P. croceum* colony/active mycelium detected.

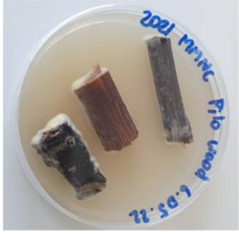

Two years

Initial mycelium in MMNC agar medium = No *P. croceum* colony detected.

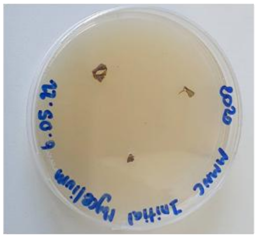

Mycelium in MMN 1/10 agar medium = No *P. croceum* colony detected.

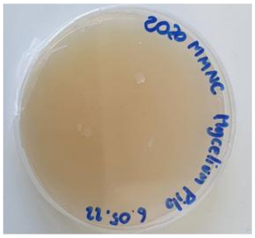

Sterile control oak wood = No *P. croceum* colony/active mycelium detected.

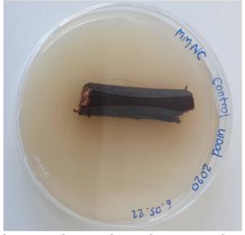

P. croceum inoculated oak wood = No *P. croceum* colony/active mycelium detected.

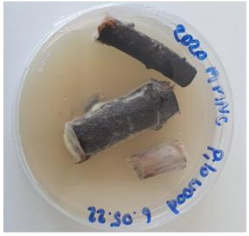

Supplementary Table S1. 14C data for the agar media used in this study

**Sample**	**^{D14}C_permil**	**err_permil**	**Remark**
Agar (MMNC)	40.0	1.9	Pre treatment agar
Agar (MMNC)	39.4	1.8	Pre treatment agar
Agar (MMN 1/10)	46.9	1.9	Treatment agar
Agar (MMN 1/10)	59.5	1.9	Treatment agar
Agar (MMN 1/10)	46.9	1.9	Treatment agar
Agar (MMN 1/10)	59.5	1.9	Treatment agar
