# Supplementary material for: Plasticity of symbiotroph-saprotroph lifestyles of Piloderma croceum associated with Quercus robur L
Source: Commun Biol. 2025 Sep 16;8:1344. doi: 10.1038/s42003-025-08762-w (PMC12441137; doi:10.1038/s42003-025-08762-w)
Supplement: Supplementary file 2 — Description of Additional Supplementary Files [file 42003_2025_8762_MOESM2_ESM.docx]

Description of Additional Supplementary Files

**File name:** Supplementary Data 1

**Description:** The source data behind the Figure1 in the paper.

**File name:** Supplementary Data 2

**Description:** The source data behind the Figure 2 in the paper.

**File name:** Supplementary Data 3

**Description:** The source data behind the Figure 3 in the paper.

**File name:** Supplementary Data 4

**Description:** Detection of *P. croceum* in the rhizosphere of oak plants after 14 weeks using Illumina sequencing.
